# Mineralocorticoid receptor antagonists for chronic central serous chorioretinopathy: systematic review and meta-analyses

**DOI:** 10.1186/s40942-022-00385-1

**Published:** 2022-06-07

**Authors:** Camila Q. Felipe, Ana Luiza Biancardi, Vinicius T. Civile, Nelson Carvas Junior, Pedro D. Serracarbassa, Marcia K. Koike

**Affiliations:** 1 Postgraduate Program in Health Sciences, Institute of Medical Care for Civil Servants in the State of São Paulo (IAMSPE), São Paulo, Brazil; 2 Laboratory of Infectious Diseases in Ophthalmology, Oswaldo Cruz Foundation (Fiocruz), Rio de Janeiro, Brazil; 3 Department of Medicine, Federal University of São Paulo, São Paulo, Brazil; 4 Cochrane Brazil and Department of Physiotherapy, Paulista University, São Paulo, Brazil; 5 Postgraduate Program in Health Sciences, IAMSPE, São Paulo, Brazil; 6 Postgraduate Program in Health Sciences, IAMSPE and Laboratory of Medical Investigation 51 (LIM-51), University of São Paulo, São Paulo, Brazil; 7 Postgraduate Program in Health Sciences of the Institute of Medical Care for Civil Servants in the State of São Paulo (IAMSPE), Avenida Ibirapuera 981, 2 andar, Vila Clementino, São Paulo, SP CEP 04029-000 Brazil

**Keywords:** Central Serous Chorioretinopathy, Mineralocorticoid Receptor Antagonists, Eplerenone, Spironolactone

## Abstract

**Background:**

Mineralocorticoid receptor antagonists (MRAs) are widely used for chronic central serous chorioretinopathy (cCSCR), but their effectiveness remains unclear. This research was conducted to evaluate the efficacy of this drugs for cCSCR.

**Methods:**

This is a review of randomized clinical trials (RCT) comparing MRAs to placebo in adults with cCSCR, using the effects of MRAs on best-corrected visual acuity (BCVA) and adverse events as primary outcomes and the effects of MRAs on anatomical parameters as secondary outcomes: central subfield thickness (CST), subretinal fluid height (SFH) and central choroidal thickness (CCT). Our all-language online search included Medline (via PubMed), Central, Embase, Lilacs, Ibecs, and RCT registers platforms, as late as May 2021. We used the Cochrane risk-of-bias tool (version 2) to assess the methodological quality of each study and synthesized the results in meta-analyses using a random-effects model.

**Results:**

The search identified 302 records, five of which were eligible, totaling 225 cCSCR patients (aged 45–62 years; M/F ratio 3.1:1) treated for 1 to 12 months with spironolactone (50 mg/day) or eplerenone (50 mg/day) *vs.* placebo. Moderate-certainty evidence suggests MRAs result in little to no improvement in BCVA compared to placebo (SMD 0.22; 95% CI − 0.04 to 0.48; studies = 5; comparisons = 6; participants = 218; I^2^ = 0%). Very low-certainty evidence suggests that, when compared to placebo, MRAs have a very uncertain impact on adverse effects (no meta-analysis was performed), and CST (MD 18.1; 95% CI − 113.04 to 76.84; participants = 145; studies = 2; I^2^ = 68%). MRAs also result in little to no difference in SFH (SMD − 0.35; 95% CI − 0.95 to 0.26; studies = 5; comparisons = 6; participants = 221; I^2^ = 76%; moderate certainty) and CCT (MD − 21.23; 95% CI − 64.69 to 22.24; participants = 206; studies = 4; comparisons = 5; I^2^ = 85%; low certainty).

**Conclusion:**

MRAs have little to no effect on BCVA. Evidence for adverse events and CST is very uncertain. MRAs also have little to no effect on SFH and CCT. These findings should be considered when prescribing MRAs for cCSCR.

This research was previous registration in the PROSPERO platform (CRD42020182601).

**Supplementary Information:**

The online version contains supplementary material available at 10.1186/s40942-022-00385-1.

## Background

Central serous chorioretinopathy (CSCR) manifests as serous detachment of the neurosensory retina, occasionally associated with retinal pigment epithelial detachment (PED). CSCR is currently considered part of the pachychoroid spectrum, a group of diseases characterized by bulging of the large choroidal vessels and thinning of the choriocapillaris and Sattler’s layer [1].

CSCR is the fourth-most common non-surgical retinal disorder, after age-related macular degeneration, diabetic retinopathy and retinal vessel occlusion [2].The incidence of CSCR is 10/100,00 in men and 1.7/100,00 in women, [3] primarily affecting men 20–60 years of age [4].

CSCR is considered chronic (cCSCR) when subretinal fluid (SRF) persists for over 3–6 months [5].The period required for chronicity has not been established, but most authors adopt a 3-month cut-off to distinguish between acute and chronic cases [6–8]. Around 13% of cCSCR patients are legally blind ten years after disease onset. In this patient population, cystoid macular degeneration, choroidal neovascularization and outer retinal atrophy are associated with increased visual loss [9].

The proposed treatment for cCSCR targets the retinal pigment epithelium (RPE), the choroidal vessels, or both, in order to enhance the ability of the RPE to remove fluid, reduce choroidal leakage or reduce fluid flow through the external blood-retinal barrier [10, 11].

Treatments like photodynamic therapy (PDT) and laser photocoagulation are invasive and may have adverse effects such as macular scarring, choroidal neovascularization and RPE atrophy [6–8, 28].

Despite the good level of safety and reasonable level of efficacy observed with the use of subthreshold micropulse laser treatment (SMPLT) for cCSCR in a number of reports [12–17], the rate of SRF resolution was lower in the PLACE trial [18] (the only randomized, prospective multicenter study of this type of treatment for cCSCR). Functional results are believed to be better when SMPLT is initiated early (before chronification) [19]. On the other hand, half-fluence PDT has been shown to be more efficient than SMPLT at SRF resolution and functional recovery following treatment for cCSCR [18] and is currently the first treatment option, notwithstanding the prohibitive cost and limited availability.

Considering the involvement of high levels of endogenous or exogenous glucocorticoids in the genesis of CSCR and the activation of mineralocorticoid receptors by glucocorticoids, some authors believe this pathway is implicated in the development of CSCR [20, 21]. There is evidence of the presence of mineralocorticoid receptors in choroidal vessels, which, when activated, induce dilatation and increase vascular permeability, resulting in SRF accumulation. Thus, mineralocorticoid receptor antagonists (MRAs), such as spironolactone and eplerenone, became an alternative treatment for cCSCR [22–29]

Parallel [27, 28] or cross-over [29] controlled clinical trials have yielded conflicting results regarding the effect of MRAs on cCSCR. The effect on functional parameters, such as best-corrected visual acuity (BCVA), ranges from moderate [21, 29] to absent [27]. A similar pattern is observed for anatomical parameters, with some studies showing improvements [23, 28, 29] and others showing no effect [27, 30].

The systematic reviews of randomized clinical trials (RCT) on the use of MRAs in cCSCR published so far have had serious limitations. A Cochrane review looking into the effects of several interventions for cCSCR [31] was published before the first RCTs on oral MRA therapy became available. Other relevant systematic reviews [32–34] pooled acute and chronic cases or pooled RCTs with other types of studies.

In the present review, we attempt to resolve the inconsistencies in the literature by employing the strictest possible methodological criteria in an evaluation of the efficacy and safety of MRAs in the treatment of cCSCR.

## Methods

This systematic review with meta-analyses was conducted in compliance with Prisma 2020 recommendations [35] and was prospectively registered on PROSPERO platform (CRD42020182601).

### Inclusion criteria

#### Types of studies

We selected RCTs with parallel or cross-over design (analyzing the first stage only) which evaluated the administration of MRAs to cCSCR patients. The sample excluded non-randomized trials, trials pooling acute and chronic forms of CSCR, cohort studies, case–control studies, and case reports.

#### Types of participants/study population

To be eligible, participants had to be adult (≥ 18 years) and diagnosed with cCSCR on optical coherence tomography (OCT), fluorescein angiography, indocyanine green angiography, or a combination of these. Studies including patients with other macular conditions (e.g., choroidal neovascularization, macular degeneration and myopic maculopathy) were not eligible.

#### Types of interventions

RCTs evaluating the effects of MRAs (spironolactone and eplerenone) on cCSCR were eligible provided at least one control group (placebo or non-intervention) was included.

#### Types of outcome parameters

The primary outcome parameters were improvement in BCVA (assessed with a ETDRS chart or similar) and adverse events (including treatment-related vision loss, retinal atrophy and choroidal neovascularization). The secondary outcome parameters were central subfield thickness (CST), subretinal fluid height (SFH) and central choroidal thickness (CCT), estimated on Spectral Domain OCT. In each study we selected the data from the longest treatment period available.

### Search strategy

The search universe included the databases Medline via PubMed (1996 to May 2021), Central (Issue 5, 2021), Embase (1974 to May 2021), Lilacs and Ibecs via VHL Regional Portal (1982 to May 2021) and three RCT platforms (https://clinicaltrials.gov, https://isrctn.com, https://ictrptest.azurewebsites.net), with no restrictions on publication language, date or status. Table 1 presents the search strategy. The search was complemented by screening the references of the selected studies and relevant systematic reviews.

**Table 1:** Search strategy

Databases	Strategies
1. *Search strategy CENTRAL*	#1. MeSH descriptor: [Central Serous Chorioretinopathy] explode all trees #2. (Central Serous Chorioretinopathies) OR (Chorioretinopathies, Central Serous) OR (Chorioretinopathy, Central Serous) OR (Serous Chorioretinopathies, Central) OR (Serous Chorioretinopathy, Central) OR (Central Serous Retinopathy) OR (Central Serous Retinopathies) OR (Retinopathies, Central Serous) OR (Retinopathy, Central Serous) OR (Serous Retinopathies, Central) OR (Serous Retinopathy, Central) #3. #1 OR #2 #4. MeSH descriptor: [Mineralocorticoid Receptor Antagonists] explode all trees #5. (Antagonists, Mineralocorticoid Receptor) OR (Receptor Antagonists, Mineralocorticoid) OR (Mineralocorticoid Antagonists) OR (Antagonists, Mineralocorticoid) OR (Aldosterone Receptor Antagonists) OR (Antagonists, Aldosterone Receptor) OR (Receptor Antagonists, Aldosterone) OR (Aldosterone Antagonists) OR (Antagonists, Aldosterone) #6. MeSH descriptor: [Spironolactone] explode all trees #7. (Spirolactone) OR (Veroshpiron) OR (Verospirone) OR (Spiractin) OR (Spirobeta) OR (Spirogamma) OR (Spirolang) OR (Spirono-Isis) OR (Spirono Isis) OR (Spironone) OR (Spirospare) OR (Aldactone) OR (Verospiron) OR (Aldactone A) OR (Aquareduct) OR (Duraspiron) OR (Espironolactona Alter) OR (Espironolactona Mundogen) OR (Flumach) OR (Frumikal) OR (Jenaspiron) OR (Novo-Spiroton) OR (Novo Spiroton) OR (NovoSpiroton) OR (Practon) OR (SC-9420) OR (SC 9420) OR (SC9420) OR (Spiro L.U.T.) OR (Spiro Von Ct) OR (Ct, Spiro Von) OR (Von Ct, Spiro) #8. MeSH descriptor: [Eplerenone] explode all trees #9. (Eplerenon) OR (Inspra) OR ("9,11- Epoxy-7-(methoxycarbonyl)-3-oxo-17- pregn-4-ene-21,17-carbolactone") #10. #4 OR #5 OR #6 OR #7 OR #8 OR #9 #11. #3 AND #10
2. *Search strategy MEDLINE* *(PubMed)*	#1. "Central Serous Chorioretinopathy"[Mesh] OR (Central Serous Chorioretinopathies) OR (Chorioretinopathies, Central Serous) OR (Chorioretinopathy, Central Serous) OR (Serous Chorioretinopathies, Central) OR (Serous Chorioretinopathy, Central) OR (Central Serous Retinopathy) OR (Central Serous Retinopathies) OR (Retinopathies, Central Serous) OR (Retinopathy, Central Serous) OR (Serous Retinopathies, Central) OR (Serous Retinopathy, Central) #2. "Mineralocorticoid Receptor Antagonists"[Mesh] OR (Antagonists, Mineralocorticoid Receptor) OR (Receptor Antagonists, Mineralocorticoid) OR (Mineralocorticoid Antagonists) OR (Antagonists, Mineralocorticoid) OR (Aldosterone Receptor Antagonists) OR (Antagonists, Aldosterone Receptor) OR (Receptor Antagonists, Aldosterone) OR (Aldosterone Antagonists) OR (Antagonists, Aldosterone) OR "Spironolactone"[Mesh] OR (Spirolactone) OR (Veroshpiron) OR(Verospirone) OR (Spiractin) OR (Spirobeta) OR (Spirogamma) OR (Spirolang) OR (Spirono-Isis) OR (Spirono Isis) OR (Spironone) OR (Spirospare) OR (Aldactone) OR (Verospiron) OR (Aldactone A) OR (Aquareduct) OR (Duraspiron) OR (Espironolactona Alter) OR (Espironolactona Mundogen) OR (Flumach) OR (Frumikal) OR (Jenaspiron) OR (Novo-Spiroton) OR (Novo Spiroton) OR (NovoSpiroton) OR (Practon) OR (SC-9420) OR (SC 9420) OR (SC9420) OR (Spiro L.U.T.) OR (Spiro Von Ct) OR (Ct, Spiro Von) OR (Von Ct, Spiro) OR "Eplerenone"[Mesh] OR (Eplerenon) OR (Inspra) OR (9,11- Epoxy-7-(methoxycarbonyl)-3-oxo-17- pregn-4-ene-21,17-carbolactone) #3. #1 AND #2
3. *Search strategy Embase (Elsevier)*	#1 'central serous retinopathy'/exp OR 'central angiospastic retinitis' OR 'central retinitis' OR 'central serous chorioretinopathy' OR 'central serous retinitis' OR 'chorioretinitis centralis serosa' OR 'retinitis centralis' OR 'retinitis centralis angiospastica' OR 'retinitis centralis serosa' OR 'retinitis, central angiospastic' OR 'retinitis, central serous' OR 'retinopathy, central serous' OR 'serous retinopathy, central' #2 'mineralocorticoid antagonist'/exp OR antimineralocorticoid OR 'mineralocorticoid receptor antagonists' OR 'spironolactone'/exp OR '17 hydroxy 7 mercapto 3 oxo 17alpha pregn 4 ene 21 carboxylic acid gamma lactone 7 acetate' OR '3 (3 oxo 7alpha acetylthio 17beta hydroxy 4 androsten 17alpha yl) propionic acid gamma lactone' OR '7alpha acetylthio 3 oxo 4 androsten 17 spiro 2 tetrahydrofuran 5 one' OR abbolactone OR acelat OR adultmin OR alaton OR alatone OR aldace OR aldactone OR 'aldactone 50' OR 'aldactone a' OR 'aldactone diurapid' OR aldopur OR aldospirone OR almatol OR aquareduct OR berlactone OR 'betaaldopur' OR carospir OR 'crl 635' OR crl635 OR diram OR duraspiron OR 'dyta urese' OR dytaurese OR flumach OR hypazon OR idrolattone OR merabis OR novospiroton OR osiren OR osyrol OR 'osyrol 50 100' OR pirolacton OR pondactone OR practon OR prilactone OR resacton OR 'sas 1060' OR sas1060 OR 'sc 9420' OR sc9420 OR spiractin OR spiridon OR spirix OR 'spiro ct' OR spiroctan OR 'spiroctan m' OR spirohexal OR spirolacton OR spirolactone OR spirolang OR spiron OR spirone OR spironex OR spirono-isis OR spironol OR spironolacton OR spironolakton OR spironone OR 'spirothiobarbiturate 03,620' OR spirotone OR 'supra puren' OR suprapuren OR uractone OR verospiron OR verospirone OR xenalon OR 'xenalon lactabs' OR youlactone OR 'eplerenone'/exp OR '9, 11alpha epoxy 4, 5 dihydro 3, 5 dioxospiro [androst 4 ene 17, 2 (3 h) furan] 7alpha carboxylic acid methyl ester' OR '9, 11alpha epoxymexrenone' OR '9alpha, 11alpha epoxy 17beta hydroxy 3 oxo 17alpha pregn 4 ene 7alpha, 21 dicarboxylic acid gamma lactone 7 methyl ester' OR '9alpha, 11alpha epoxymexrenone' OR 'cgp 30 083' OR 'cgp 30,083' OR cgp30083 OR elecor OR epoxymexrenone OR inspra OR 'mexrenone, 9, 11alpha epoxy' OR 'sc 66,110' OR sc66110 #3 [embase]/lim NOT ([embase]/lim AND [medline]/lim) #1 AND #2 AND #3
4. *Search strategy Lilacs e Ibecs* *(Portal Regional BVS)*	#1. MH:"Coriorretinopatia Serosa Central" OR (Coriorretinopatia Serosa Central) OR (Coriorretinopatía Serosa Central) OR (Central Serous Chorioretinopathy) OR (Central Serous Chorioretinopathies) OR (Central Serous Retinopathies) OR (Central Serous Retinopathy) OR (Chorioretinopathies, Central Serous) OR (Chorioretinopathy, Central Serous) OR (Retinopathies, Central Serous) OR (Retinopathy, Central Serous) OR (Serous Chorioretinopathies, Central) OR (Serous Chorioretinopathy, Central) OR (Serous Retinopathies, Central) OR (Serous Retinopathy, Central) OR MH:C11.768.175$ #2. MH:"Antagonistas de Receptores de Mineralocorticoides" OR (Mineralocorticoid Receptor Antagonists) OR (Antagonistas de Receptores de Mineralocorticoides) OR (Antagonistas da Aldosterona) OR (Aldosterone Antagonists) OR (Aldosterone Receptor Antagonists) OR (Antagonists, Aldosterone) OR (Antagonists, Aldosterone Receptor) OR (Antagonists, Mineralocorticoid) OR (Antagonists, Mineralocorticoid Receptor) OR (Mineralocorticoid Antagonists) OR (Receptor Antagonists, Aldosterone) OR (Receptor Antagonists, Mineralocorticoid) OR MH:D06.347.700$ OR MH:D27.505.696.399.450.600$ OR MH:D27.505.696.560.500.726.249$ OR MH:Espironolactona OR Espironolactona OR Spironolactone OR (Espirolactona) OR Aldactone OR (Aldactone A) OR Aquareduct OR (Ct, Spiro Von) OR Duraspiron OR (Espironolactona Alter) OR (Espironolactona Mundogen) OR Flumach OR Frumikal OR Jenaspiron OR (Novo Spiroton) OR Novo-Spiroton OR NovoSpiroton OR Practon OR (SC 9420) OR SC-9420 OR SC9420 OR Spiractin OR (Spiro L.U.T.) OR (Spiro Von Ct) OR Spirobeta OR Spirogamma OR Spirolactone OR Spirolang OR (Spirono Isis) OR Spirono-Isis OR Spironone OR Spirospare OR Veroshpiron OR Verospiron OR Verospirone OR (Von Ct, Spiro) OR MH:D02.540.679$ OR MH:D04.210.500.745.745.855$ OR MH:Eplerenona OR Eplerenona OR Eplerenone OR (9,11-Epoxi-7-(Metoxicarbonil)-3-Oxo-17-Pregn-4- eno-21,17-Carbolactona) OR Inspra OR (9,11-Epoxy-7-(methoxycarbonyl)-3- oxo-17-pregn-4-ene-21,17-carbolactone) OR Eplerenon OR MH:D02.540.383$ OR MH:D04.210.500.745.745.329$ #1 AND #2
5. *Search strategy Clinicaltrials.gov*	Central Serous Chorioretinopathy AND Mineralocorticoid Receptor Antagonists OR Spironolactone OR Eplerenone
6. *Search strategy WHO ICTRP*	"Central Serous Chorioretinopathy" OR "Central Serous Retinopathy" AND "Mineralocorticoid Receptor Antagonists" OR "Mineralocorticoid Antagonist" OR "Spironolactone" OR "Eplerenone"
7. *Search strategy* ISRCTN	(Central Serous Chorioretinopathy) OR (Central Serous Retinopathy) AND (Mineralocorticoid Receptor Antagonists) OR (Mineralocorticoid Antagonist) OR (Spironolactone) OR (Eplerenone)

### Study selection

The software Rayyan [36] allowed the team of reviewers to remove duplicates of the selected publications and manage the sample with transparency. The evaluation of eligibility was performed independently by two reviewers (VTC and NCJ). First, the studies were considered for inclusion based on their titles and abstracts. Selected publications were then submitted to full—text analysis. In case of disagreement, a third reviewer (CQ) was consulted and a consensus was reached. When necessary, the original authors were contacted for clarification.

### Data extraction

Two reviewers (VTC and NCJ) independently retrieved the data and results of each publication, consulting a third reviewer (CQ) in the case of divergence. When further data were deemed necessary, the original authors were contacted.

The extracted data covered methodology (design, units of randomization and analysis), study population (country, number of subjects, age, sex, inclusion/exclusion criteria), interventions (number of subjects randomized for each group, drug name, dosage, frequency and route, and duration of treatment), and outcome parameters (see section above). Table 2 shows the main aspects of each study.

**Table 2:** Characteristics of included studies

Study	Duration of treatment	Study design	Participants	Dose (mg/day)	Previous steroids	Symptom duration (mean)	Sponsorship source	Conflicts of interest
Bousquet 2015	1 month	ECR / crossover	Spironolactone Group (n = 8) [n = 7 male and n = 1 female] with mean of age 48 years old and Placebo Group (n = 7) [n = 5 male and n = 2 female] with mean of age 44,7 years old	Spironolacotone group 50 mg / Placebo group 50 mg, once a day (tablets)	Sprinonolactone Group (n = 3) and Placebo Group (n = 2)	Spironolactone Group 12.3 months and Placebo Group (n = 7.4)	Inserm provided financial support for the statistical analysis and was promoter of the study	F. Behar-Cohen and N. Farman are inventors on a patent protecting the use of MR antagonists for retinal edema. The patent rights belong to Inserm. None of the remaining authors have any conflicting interests to disclose
Pichi 2016	1 month (One week + 3 weeks)	ECR / crossover	Spironolactone Group (n = 20); Eplerone Group (n = 20); Placebo Group (n = 20); n = 46 men and 14 women with overall mean age of 51.1 years old	Spironolactone dose (25 mg + 50 mg) / Placebo dose (25 mg + 50 mg) / Eplerone (dose (25 mg + 50 mg), once a day (tablets)	22 of 60 participants	–	Not informed	No conflicting relationship exists for any author
Schuwartz 2017	6 months (treatment for 3 months) / One week + 11 weeks	ECR / parallel	Eplerone Group (n = 12) [n = 8 male and n = 4 female] with mean of age 50.6 years old / Placebo Group (n = 5) [n = 4 male and n = 1 female] with mean of age 47.2 years old	Eplerone dose (25 mg + 50 mg) and Placebo dose (25 mg + 50 mg), once a day (tablets)	Eplerone Group (n = 3) and Placebo Group (n = 2)	Eplerone Group 3.1 years and Placebo Group 2.2 years	No financial support was given for this study	None declared
Rahimy 2018	9 weeks (one week + 8 weeks	ECR / parallel	Eplerone Group (n = 10) [n = 8 male and n = 2 female] with mean of age 50 years old / Placebo Group (n = 5) [n = 4 male and n = 1 female] with mean of age 62.2 years old	Eplerone dose (25 mg + 50 mg) and Placebo dose (25 mg + 50 mg), once a day (tablets)	–	–	Supported, in part, by an Innovation Grant awarded through Wills Eye Hospital. The funding organization had no role in the design or conduct of the study; management, analysis, or interpretation of the data; preparation, review, or approval of the manuscript; and decision to submit the manuscript for publication	None of the authors has any conflicting interests to disclose
Lotery 2018	12 months (one week + 47 weeks)	ECR / parallel	Eplerone Group (n = 57) [n = 42 male and n = 15 female] with mean of age 47.4 years old / Placebo Group (n = 54) [n = 43 male and n = 14 female] with mean of age 49.9 years old	Eplerone dose (25 mg + 50 mg) and Placebo dose (25 mg + 50 mg), once a day (tablets)	Eplerone Group (n = 12) and Placebo Group (n = 15)	Eplerone Group 8 months (median) and Placebo Group 9 months (median)	This project was funded by the Efficacy and Mechanism Evaluation Programme (13/94/15), which is a Medical Research Council and National Institute for Health Research partnership. The views expressed in this publication are those of the authors and do not necessarily reflect those of the Medical Research Council, National Institute for Health Research, or the Department of Health and Social Care. The funder and the sponsor of the study had no role in study design, data collection, data analysis, data interpretation or writing of the report. The corresponding author had full access to all the data in the study and had final responsibility for the decision to submit for publication	AL reports speaker fees from, and has attended advisory board meetings of, Novartis, Bayer, Roche, Allergan, Gyroscope Therapeutics, and Boehringer Ingelheim. SS reports research grants and speaker fees from, and has attended advisory board meetings of, Novartis, Bayer, Roche, Allergan, Optos, Heidelberg Engineering, and Boehringer Ingelheim. FB-C is an inventor on a patent protecting the use of mineralocorticoid receptor antagonists for retinal oedema. TP reports research grants and speaker fees from, and has attended advisory board meetings of, Novartis, Bayer, Roche, Optos, Heidelberg Engineering, Welch Allyn, and Boehringer Ingelheim. All other authors declare no competing interests

### Assessment of risk of bias and certainty of evidence

Two reviewers (VTC and NCJ) independently assessed the risk of bias (using the Cochrane risk-of-bias tool version 2-RoB2) and the certainty of evidence in the selected studies, following the above procedure in the case of divergence.

The RoB2 covers the following dimensions: bias arising from the randomization process, bias due to deviations from the intended interventions, bias due to missing outcome data, bias in the measurement of the outcome, and bias in the selection of reported results. The study was focused on the ‘assignment to intervention’ (or ‘intention-to-treat’) effect at baseline. Within each domain, responses to signaling questions were either ‘yes’, ‘probably yes’, ‘no’, ‘probably no’ or ‘no information’. The final risk-of-bias judgment of the algorithm was ‘low risk of bias’, ‘some concerns’ or ‘high risk of bias’. The overall risk of bias of each result corresponded to that of the least favorable assessment in all domains.

We evaluated the certainty of evidence for each outcome with the software GRADEpro GDT. Certainty was downgraded by one level for serious limitations and by two levels for very serious limitations, based on predefined criteria (study limitations, inconsistency, indirectness, imprecision of estimates, and presence of publication bias). The final result fell into one of the following categories: high certainty, moderate certainty, low certainty, and very low certainty.

### Measures of treatment effect and data synthesis

To conduct meta-analyses we employed the software Review Manager (RevMan 5.4, Copenhagen: The Nordic Cochrane Centre, The Cochrane Collaboration, 2021), combining the effects in random effects models with the inverse-variance method or, when necessary, the generic inverse-variance method. We expressed the effects as mean difference (MD) and their respective 95% confidence intervals (95% CI) or standardized mean difference (SMD) and their respective intervals, grouping the studies according to the criteria of eligibility of each outcome. A minimal clinically important difference (MCID) was an improvement in BCVA of at least 5 letters on the ETDRS chart (or equivalent) or a 10% reduction in CST, SFH and CCT. We assessed the heterogeneity using Cochran`s Q and I^2^ tests and visual inspection of the forest plots. When the heterogeneity was important, accompanied by a statistical significance (P < 0.10), we investigated the possible reason through subgroup analysis according to treatment duration (< 3 months *vs* ≥ 3 months).

### Sensitivity analysis and assessment of reporting bias

The sensitivity analysis used to assess the robustness of the data excluded studies with high risk of bias (or some concerns), unpublished studies and studies influenced by funding.

Finally, we planned to evaluate the influence of publication bias on the results using funnel plots and Egger’s test, provided each meta-analysis included at least 10 studies.

### Availability of data and materials

The dataset supporting the conclusions of this article is included within the article and its Additional file 1: Data Extraction and Additional file 2.

## Results

**Fig. 1:**
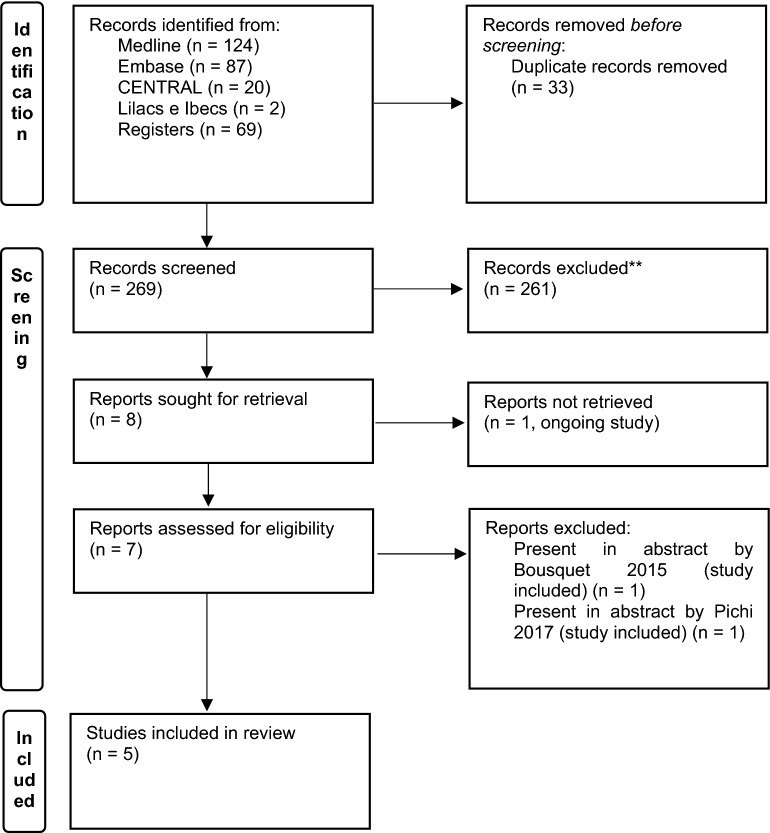
Selection of eligible studies and reasons for exclusion (Prisma 2020 Flow Diagram)

The search yielded 233 records from the databases Medline (n = 124), Central (n = 20), Embase (n = 87) and Lilacs/Ibecs (via VHL Regional Portal) (n = 2), in addition to 69 records from specialized platforms. The elimination of duplicates left 269 records for title and abstract analysis, of which 261 were excluded, leaving 8 full articles for eligibility assessment. We subsequently excluded two studies because they included abstracts presented at events related to two studies already included and one study which was ongoing at the time of writing. The screening process left a final sample of 5 RCTs (all of which contained at least one meta-analysis) for qualitative and quantitative synthesis. Figure 1 shows the steps in the triage and inclusion of studies.

### Studies included in the analysis

Table 2 shows the characteristics of the 5 studies included in the final sample: three two-armed, parallel-design RCTs, [27, 28, 30] one two-armed cross-over RCT, [23] and one three-armed RCT [29]. One study was multicenter; [30] the remainder single-center [23, 27–29]. Treatment lasted 1 month, [23, 29] 2 months, [28] 5 months, [27] or 12 months [30]. Prepublished protocols were available for 4 studies.

Taken together, the 5 studies had 225 randomized participants, 221 of whom were analyzed regarding at least one of the outcome parameters. All studies included participants with cCSCR (‘chronic’ being defined as the presence of subretinal fluid for over 3 months). The mean age was 47–51 years for the groups treated with MRAs and 45–62 years for the control groups. The male/female ratio was 3.1:1.

Two studies [23, 29] compared spironolactone to placebo in pill form: 25 mg/day in the first week, 50 mg/day from the second week on (total: 1 month). Four studies [27–30] compared eplerenone to placebo in pill form: 25 mg/day in the first week, 50 mg/day from the second week on (total: 1–12 months). One study [29] (three-armed RCT) evaluated both drugs.

### Risk of bias in the included studies

Three studies [23, 29, 30] completed the intended interventions with no deviations in any of the outcome parameters. Four studies performed adequate analyses based on intention to treat approach (or intention to treat with adjustments for missing data). One study [28] did not use an intention-to-treat approach and presented incomplete outcome data.

All studies presented the outcomes adequately, with the examiners blinded at all times (= low risk of bias).

Only one study [30] had an adequate plan of analysis for all outcomes (low risk of bias). No plan of analysis existed for most of the outcomes in the protocols of the other studies (= some concerns, or high risk of bias).

**Fig. 2:**
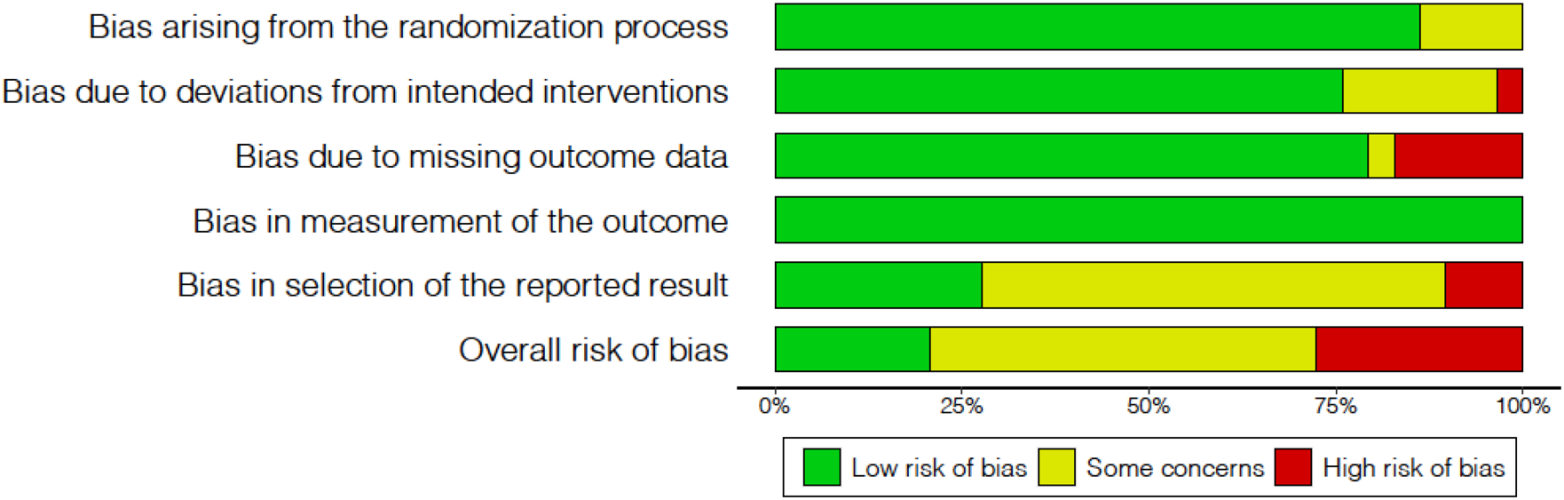
Risk of bias: authors’ judgements of each type of bias, expressed in percentages.

Only one study [30] had a low risk of bias overall for all outcomes. In one study, [27] the overall assessment was ‘some concerns’ for all outcomes, while another [28] displayed a high risk of bias for all outcomes in the overall risk assessment. One study [23] presented 'some concerns' for most outcomes, while another [29] presented high risk of bias for the outcomes 'macular thickness' and 'subfoveal choroidal thickness'. Figure 2 summarizes the risk of bias in each domain.

### Publication bias

Since the number of RCTs included in the analysis was smaller than 10, we were unable to evaluate the influence of publication bias on the results (using funnel plots and Egger’s test), as originally intended.

### Results of meta-analyses

#### BCVA

**Fig. 3:**
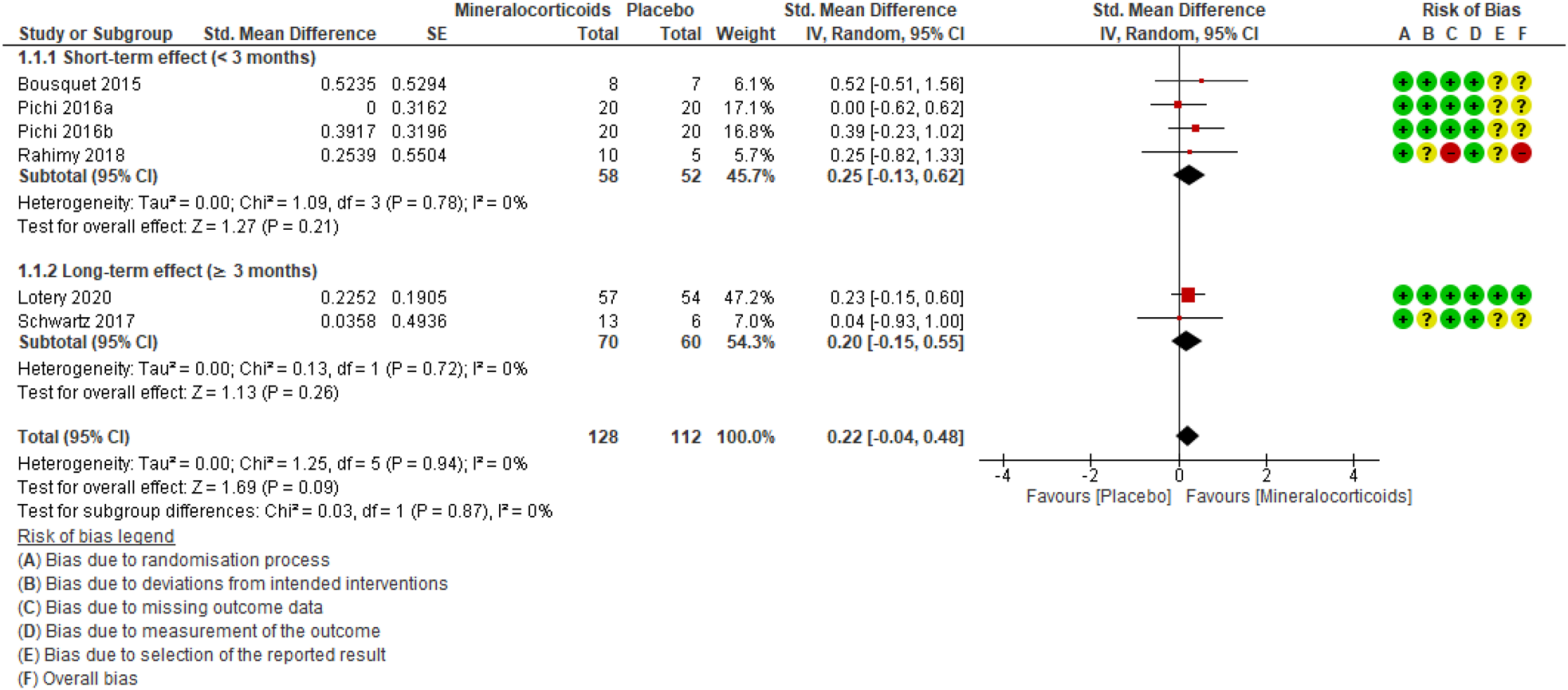
Forest plot of comparison: MRAs vs placebo. Outcome: BCVA.

The five included studies [23, 27–30] totaled 218 participants for this outcome. MRAs probably have little to no positive effect on BCVA (evaluated with the ETDRS chart or the Snellen chart and expressed in letter counting or logarithmic minimum angle of resolution) when compared to placebo. The effect ranged from ‘clinically unimportant improvement’ to ‘clinically unimportant worsening’ (SMD = 0.22; 95% CI − 0.04 to 0.48; studies = 5; comparisons = 6; I^2^ =0%) (Fig. 3).

On a scale from 0 to 100 letters (ETDRS chart; more letters = better BCVA), acuity improved by 0.99 letters in patients treated with MRAs (95% CI 0.18 fewer to 2.16 more letters; 1% absolute improvement [95% CI 0.2% worse to 2.2% better]; 1.27% relative improvement (95% CI 0.23% worse to 2.77% better; MCID = 5 letters). Applying the GRADE criteria, there is evidence of moderate certainty that MRAs have little (clinically unimportant) to no positive effect on BCVA when compared to placebo (evidence downgraded due to imprecision) (Table 3).

**Table 3:** Summary of findings

Outcomes	Anticipated absolute effects* (95% CI)		Relative effect (95% CI)	№ of participants (studies)	Certainty of the evidence (GRADE)	Comments
	Risk with Placebo	Risk with Mineralocorticoids (Spironolactone or Eplerenone)				
Best-corrected visual acuity (BCVA) assessed with: Early Treatment Diabetic Retinopathy Study (ETDRS) chart (more = better) follow up: range 1 month to 12 months	The mean bestcorrected visual acuity was 79.5 letters	MD 0.99 letters more (0.18 fewer to 2.16 more)	–	218 (5 RCTs)	⨁⨁⨁◯ MODERATE a	Mineralocorticoid receptor antagonists likely results in little to no difference in bestcorrected visual acuity Absolute percent difference = 1.0% absolute improvement (95% CI 0.2% worsening to 2.2% improvement) Relative percent difference = 1.27% relative improvement (95% CI 0.23% worsening to 2.77% improvement) SMD = -0.22 (95% CI -0.04 to 0.48) MCID = 5 letters
Subretinal fluid height assessed with: Optical Coherence Tomography (fewer = better) follow up: range 1 month to 12 months	TThe mean subretinal fluid thickness was 72.5 micrometers	MD 2.17 micrometers fewer (5.89 fewer to 1.61 more)	–	243 (5 RCTs)	⨁⨁⨁◯ MODERATE ^a,b^	Mineralocorticoid receptor antagonists likely results in little to no difference in subretinal fluid height Absolute percent difference = not applicable Relative percent difference = 1.82% relative improvement (95% CI 1.35% worsening to 4.95% improvement) SMD = -0.35 (95% CI -0.95 to 0.26) MCID = 10%
Subfoveal choroidal thickness assessed with: Optical Coherence Tomography (fewer = better) follow up: range 1 month to 12 months	The mean subfoveal choroidal thickness ranged from 379.5-527 micrometers	MD 21.23 micrometers fewer (64.69 fewer to 22.24 more)	–	228 (4 RCTs)	⨁⨁◯◯ LOW a,c,d	Mineralocorticoid receptor antagonists may result in little to no difference in subfoveal choroidal thickness Absolute percent difference = not applicable Relative percent difference = 4.6% relative improvement (95% CI 4.8% worsening to 14% improvement) MCID = 10%
Central macular thickness assessed with: Optical Coherence Tomography (fewer = better) follow up: range 2 months to 12 months	3The mean central macular thickness ranged from 270- 380 micrometers	MD 18.1 micrometers fewer (113.04 fewer to 76.84 more)	–	145 (3 RCTs)	⨁◯◯◯ VERY LOW ^e,f,g^	The evidence is very uncertain about the effect of mineralocorticoids on central macular thickness Absolute percent difference = not applicable Relative percent difference = 4.9% relative improvement (95% CI 20.8% worsening to 30.6% improvement) MCID = 10%

GRADE Working Group grades of evidence

High certainty: We are very confident that the true effect lies close to that of the estimate of the effect

Moderate certainty: We are moderately confident in the effect estimate: The true effect is likely to be close to the estimate of the effect, but there is a possibility that it is substantially different

Low certainty: Our confidence in the effect estimate is limited: The true effect may be substantially different from the estimate of the effect

Very low certainty: We have very little confidence in the effect estimate: The true effect is likely to be substantially different from the estimate of effect

Mineralocorticoid receptor antagonists (Spironolactone or Eplerenone) compared to placebo for chronic central serous chorioretinopathy

Patient or population: Chronic central serous chorioretinopathy

Setting: Outpatient

Intervention: Mineralocorticoid receptor antagonists (Spironolactone or Eplerenone)

*CI* Confidence interval, *MD* Mean difference, *RCT* Randomised controlled trial, *SMD* Standardised mean difference, *MCID* Minimal clinically important difference

Downgraded one level by imprecision. Less than 400 participants were included in the meta-analysis.

We found high heterogeneity (I^2^ = 79%) but we explained the inconsistency by subgroup analysis.

Downgraded one level by study limitations (risk of bias). We performed a sensitivity analysis for high overall risk of bias (due to selection of the reported) result caused by Pichi et al. (three-arm trial). We found an important change on pooled result.

We found high heterogeneity (I^2^ = 85%) but we explained the inconsistency by subgroup analysis.

Downgraded one level by study limitations (risk of bias). We performed a sensitivity analysis for high overall risk of bias (due to missing outcome data) caused by Rahimy et al. We found an important change on pooled result.

We found high heterogeneity (I^2^ = 68%) but we explained the inconsistency by subgroup analysis.

Downgraded two levels by imprecision. Less than 400 participants were included in the meta-analysis, and the absolute effect varied from an important clinically benefit to an important clinically harm.

^*^The risk in the intervention group (and its 95% confidence interval) is based on the assumed risk in the comparison group and the relative effect of the intervention (and its 95% CI)

### Adverse events

The five included studies [23, 27–30] totaled 225 participants for this outcome. When compared to placebo, the evidence for a positive effect of MRAs on adverse events is very uncertain. Applying the GRADE criteria, the evidence for this outcome is of very low certainty (evidence downgraded one level due to risk of bias and two levels due to imprecision) (Table 3).

Since the selected studies did not consistently report adverse events, no meta-analysis was performed for this outcome.

### Central subfield thickness (CST)

**Fig. 4:**
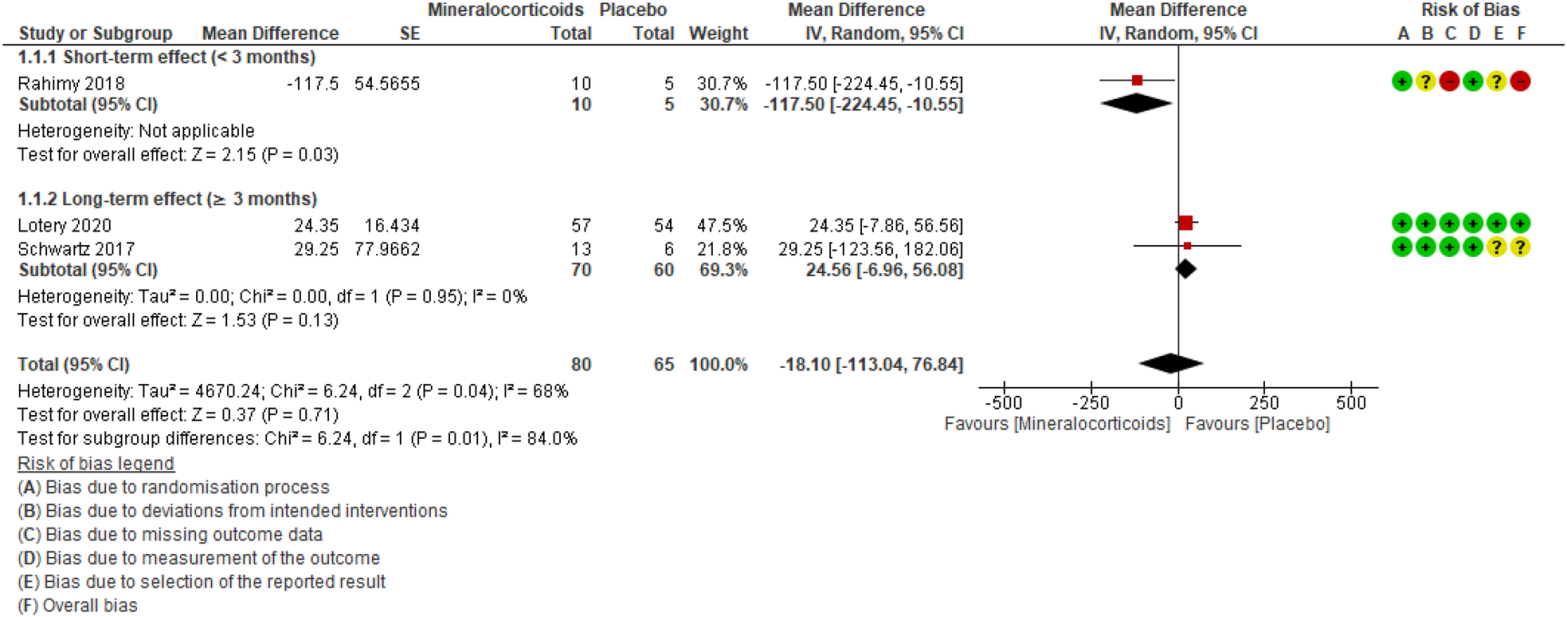
Forest plot of comparison: MRAs vs placebo. Outcome: CST.

The three studies [27, 28, 30] evaluating CST totaled 143 participants. The evidence of MRA-induced reduction in CST on OCT (smaller = better) when compared to placebo is very uncertain, ranging from clinically important improvement to clinically important worsening (MD -18.1 [95% CI − 113.04 to 76.84]; studies = 3; I^2^ = 68%; absolute percentage difference not applicable [data not expressed on a scale]; relative percentage improvement = 4.9% [95% CI 20.8 worse to 30.6 better]; MCID = 10%) (Fig. 4). Applying the GRADE criteria, the evidence for CST reduction was of very low certainty, compared to placebo (evidence downgraded one level due to risk of bias and two levels due to imprecision) (Table 3).

We also analyzed a subgroup of participants treated with MRA for less than 3 months (short-term effect). When compared to controls, the MRA group displayed a 117.5 μm reduction in CST (95% CI 224.45 smaller to 10.55 smaller; participants = 15; studies = 1; I^2^ = not applicable; absolute percentage difference not applicable; relative percentage improvement = 34.1% [95% CI 3.1% better to 65.1% better]; MCID = 10%). Applying the GRADE criteria, the evidence for MRA-induced CST reduction in up to 3 months of treatment is very uncertain when compared to placebo (evidence downgraded one level due to risk of bias and two levels due to imprecision). In the subgroup of participants treated for more than 3 months (long-term effects), CST increased by 24.56 μm (95% CI − 6.96 smaller to 56.08 bigger; participants = 130; studies = 2; I^2^ = 0%; absolute percentage difference not applicable; relative percentage worsening = 7.4% [95% CI 16.9% worse to 2.1% better]; MCID = 10%). Applying the GRADE criteria, the evidence that over 3 months of treatment with MRA results in little to no reduction in CST when compared to placebo is uncertain (evidence downgraded two levels due to imprecision).

With the exclusion of one study, [28] the sensitivity analysis modified the estimated effect of MRAs from a slight reduction to a slight increase in CST, when compared to placebo (MD 24.56 [95% CI − 6.96 to 76.84]; participants = 130; studies = 2; I^2^ = 0%).

### Subretinal fluid height (SFH)

**Fig. 5:**
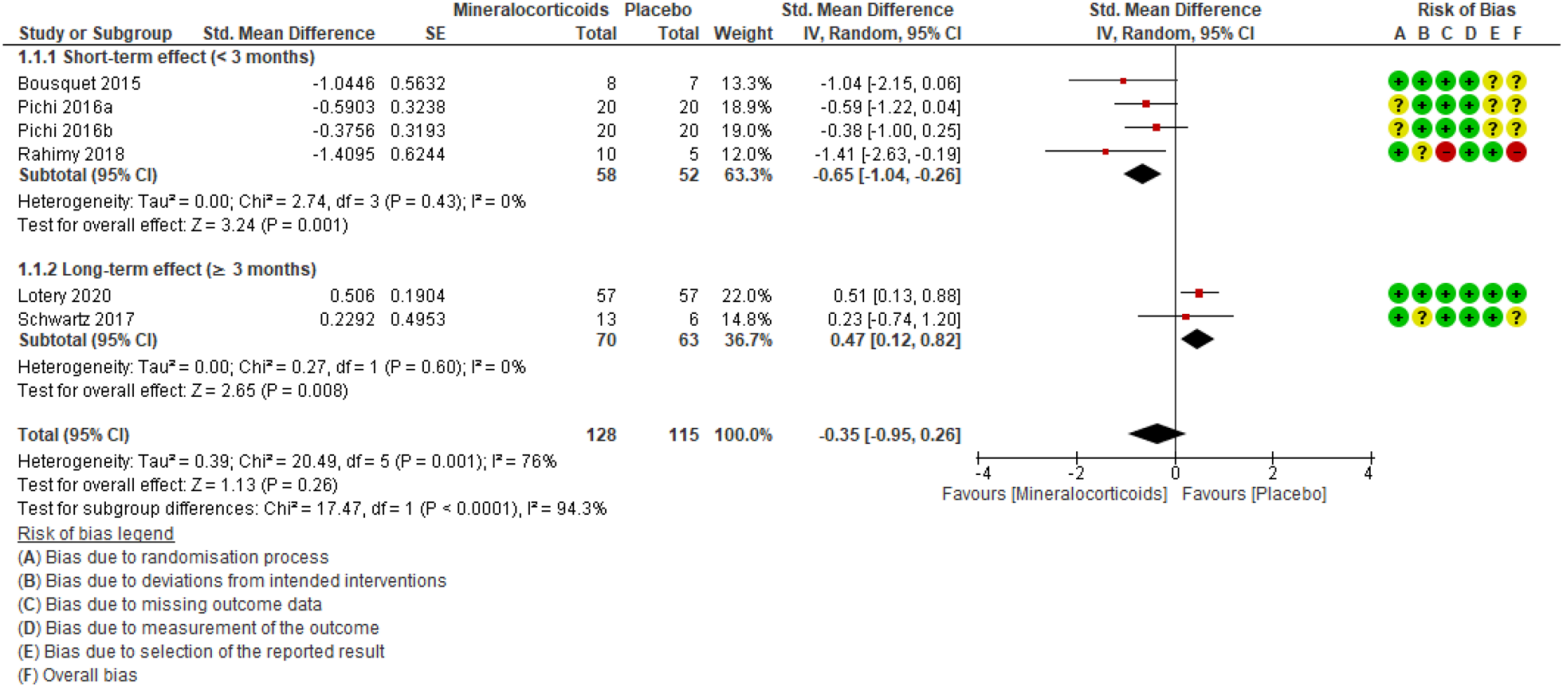
Forest plot of comparison: MRAs vs placebo. Outcome: SFH.

Five studies [23, 27–30] totaling 221 participants evaluated this outcome. One study compared spironolactone to placebo, three studies compared eplerenone to placebo, and one study tested both drugs in relation to placebo. When compared to placebo, MRAs probably have little to no effect on SFH reduction on OCT (smaller = better), ranging from clinically unimportant improvement to clinically unimportant worsening (SMD -0.35 [95% CI − 0.95 to 0.26]; studies = 5; comparisons = 6; I^2^ = 76%) (Fig. 5).

On OCT (smaller = better), the SFH of participants treated with MRA decreased by 2.17 μm (95% CI − 5.89 less to 1.61 more; absolute percentage difference not applicable [data not expressed on a scale]; relative percentage improvement = 1.82% [95% CI 1.35% worse to 4.95% better]; MCID = 10%). Applying the GRADE criteria, the evidence that MRAs have little to no effect on SFH is moderately certain, when compared to placebo (evidence downgraded one level due to imprecision) (Table 3).

We also analyzed a subgroup of participants treated with MRA for less than 3 months (short-term effect). When compared to controls, the MRA group displayed a 57.72 μm reduction in SFH (95% CI 92.35 smaller to 23.1 smaller; participants = 90; studies = 3; comparisons = 4; I^2^ = 0%; absolute percentage difference not applicable; relative percentage improvement = 42.5% [95% CI 17% better to 68% better]; MCID = 10%). Applying the GRADE criteria, we found moderately certain evidence that treatment with MRAs for up to three months probably reduces SFH, when compared to placebo (evidence downgraded one level due to imprecision). The subgroup of participants treated for over 3 months (long-term effect) displayed a 2.9 μm increase in SFH (95% CI 0.74 greater to 5.1 greater; participants = 133; studies = 2; I^2^ = 0%; absolute percentage difference not applicable; relative percentage worsening = 0.02% [95% CI 0.01% worse to 0.04% worse]; MCID = 10%). Applying the GRADE criteria, the evidence that treatment with MRA for over 3 months produces little to no increase in SFH, when compared to placebo, is moderately certain (evidence downgraded one level due to imprecision).

With the exclusion of one study, [28] the sensitivity analysis confirmed that MRAs probably cause little to no change in SFH, in relation to placebo (MD − 1.2 [95% CI − 4.8 to 2.5]; participants = 228; studies = 4; comparisons = 3; I^2^ = 74%).

### Central choroidal thickness (CCT)

**Fig. 6:**
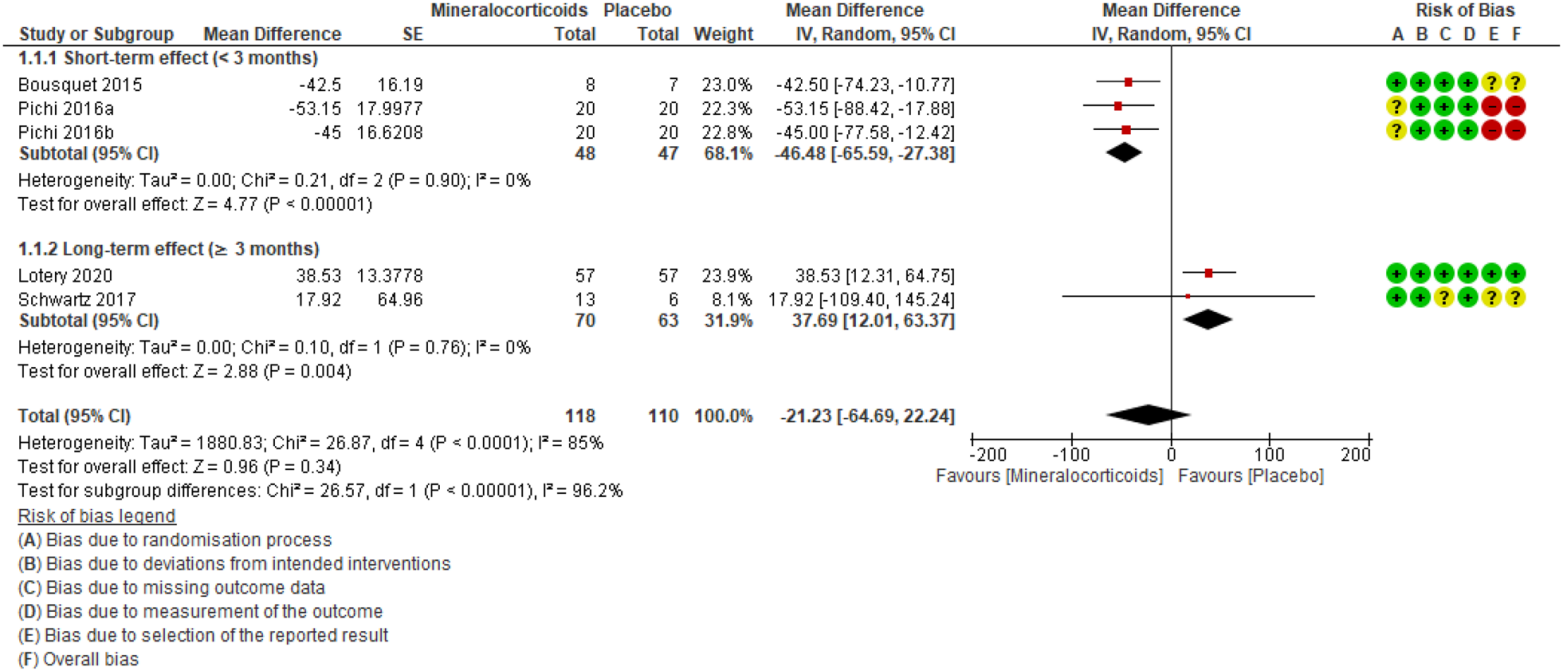
Forest plot of comparison: MRAs vs placebo. Outcome: CCT.

Four studies [23, 27, 29, 30] totaling 206 participants evaluated this outcome. One study compared spironolactone to placebo, two studies compared eplerenone to placebo, and one study evaluated both drugs in relation to placebo. When compared to placebo, MRAs may have little to no effect on CCT reduction on OCT (smaller = better), ranging from clinically important improvement to clinically unimportant worsening (MD − 21.23 [95% CI − 64.69 to 22.24; studies = 4; comparisons = 5; I^2^ = 85%; absolute percentage difference not applicable [data not expressed on a scale]; relative percentage of improvement = 4.6% [95% CI 4.8% worse to 14% better]; MCID = 10%) (Fig. 6). Applying the GRADE criteria, the evidence that MRAs result in little to no difference in CCT is of low certainty, when compared to placebo (evidence downgraded one level due to risk of bias and one level due to imprecision) (Table 3).

We also analyzed a subgroup of participants treated with MRA for less than 3 months (short-term effect). When compared to controls, the MRA group displayed a 46.48 μm reduction in CCT (95% CI 65.59 smaller to 27.38 smaller; participants = 75; studies = 2; comparisons = 3; I^2^ = 0%; absolute percentage difference not applicable; relative percentage of improvement = 10% [95% CI 5.9% better to 14% better]; MCID = 10%). Applying the GRADE criteria, the evidence that up to 3 months of treatment with MRAs reduces CCT slightly in comparison to placebo was moderately certain (evidence downgraded one level due to imprecision). The subgroup of participants treated for over three months (long-term effect) displayed a 37.69 μm increase in CCT (95% CI 12.01 greater to 63.37 greater; participants = 133; studies = 2; I^2^ = 0%; absolute percentage difference not applicable; relative percentage of worsening = 8.2% [95% CI 2.6% worse to 13.8% worse]; MCID = 10%). Applying the GRADE criteria, the evidence that over 3 months of treatment with MRAs results in increased CCT when compared to placebo is moderately certain (evidence downgraded one level due to imprecision).

With the exclusion of one study, [28] the sensitivity analysis modified the estimated effect of MRAs, from little or no reduction in CT to little or no increase in CCT, when compared to placebo (MD 1.94 [95% CI − 65.54 to 69.41]; participants = 148; studies = 3; I^2^ = 87%).

## Discussion

To our knowledge, this is the first systematic review with meta-analyses focusing exclusively on RCTs evaluating clinical response to MRA in the treatment of cCSCR.

The included studies show that eplerenone and spironolactone affect BCVA in cCSCR patients ranging from clinically unimportant worsening to clinically unimportant improvement (moderate certainty; downgrading due to imprecision). The effect of MRAs on CST is very uncertain (downgrading due to risk of bias, inconsistency and imprecision). Likewise, MRAs result in little to no change in SFH (moderate certainty; downgrading due to imprecision) or CCT (low certainty; downgrading due to risk of bias and imprecision).

All the RCTs included in this review investigated the effect of MRAs *vs* placebo in treatments lasting 1–12 months, using dosages of 25–50 mg/day. In clinical practice the minimum duration of treatment with these drugs has not been established, but the fact that most physicians prescribe them for three or more months at a dosage of 25–50 mg/day suggests the reviewed RCTs are representative of actual clinical practice. The male/female proportion in the five RCTs (3.1:1) was lower than in most other studies, [3, 4] but the age range (47–51 years) was compatible with the literature [3, 4].

Although the reviewed RCTs were considered to have low risk of bias in most domains, the meta-analyses for CST, SFH and CCT displayed high levels of heterogeneity, possibly due to the wide variation in treatment duration (1–12 months). In fact, anatomical outcomes were generally better in studies with shorter periods of treatment (1–2 months) [23, 28, 29].

A Cochrane systematic review with meta-analyses [31] published in 2010 investigated the efficacy of available treatments for cCSCR but did not include comparisons between MRAs and placebo, as in the present study. The earliest studies making such comparisons [22–24] were published after 2010.

Much controversy exists in the literature regarding the use of MRAs as an alternative treatment for cCSCR [22–30, 32].

The most recent systematic reviews with meta-analyses [32–34] pooled acute and chronic cases of CSCR. However, the inclusion of acute cases may confound the analysis of the efficacy of MRAs due to the high likelihood of anatomical and functional recovery of patients with the acute form, even without interventions [37–39]. We therefore only reviewed RCTs focused on the chronic form. Another systematic review with meta-analysis [34] from 2018 was conducted before the publication of the Vici Trial [30], which currently has the lowest risk of methodological bias of any RCT on the subject, thus with a possible potential impact on the statistical analysis. Finally, a Chinese systematic review with meta-analysis [33] included both RCTs and observational studies, without segregating them in the results. This fact may have contributed to the appearance of a positive effect of MRAs on anatomical parameters (SFH and CCT).

Improvement in BCVA in CSCR patients requires the recovery of the macular architecture and photoreceptor function, once the SRF has been absorbed [9]. Some recent studies have shown the existence of functional damage in the first months after SRF accumulation, with potential negative impacts on the patients quality of life, [40] raising the issue of the benefit of early onset of therapy [4, 41, 42] for patients with CSCR. The absence of clinically important improvement in BCVA in the current review may be explained by the fact that chronic CSCR patients treated with MRAs experience little to no reduction in SFH.

In this review we conducted a comprehensive search in major databases and RCT platforms, identifying both published and unpublished studies, thereby minimizing the risk of publication bias. On the other hand, the small number of RCTs included in the quantitative synthesis (n = 5) made it impossible to statistically verify the presence of publication bias.

One of the challenges encountered in this review was the high level of heterogeneity in three of the meta-analyses. We did not evaluate heterogeneity by analyzing preplanned subgroups (age, sex, type of personality, corticoid use, gastric infection with *Helicobacter pylori*, smoking, sleep and circadian rhythm disorders, exogenous use of testosterone, occupational activity) due to the absence of specific results, but when the participants were segregated according to time of treatment, the inconsistency was explained.

Reliance on only 5 RCTs with a small number of participants and without sample size calculation (with one exception) [30] may have influenced the estimates of our meta-analyses. Moreover, the non-standardized cross-over design of two of the studies [23, 29] (one of which was three-armed) may have biased the summary estimate due to carry-over effects.

## Conclusions

In view of the multifactorial nature of CSCR [43] and the prohibitive cost of state-of-the-art therapies like PDT, [6–8, 37] treating patients with the chronic form can be challenging. Over the last few years, MRAs have emerged as a safe and accessible alternative, although the efficacy of these drugs remains uncertain [22, 24–30, 32].

This review found that MRAs (spironolactone and eplerenone) have little to no effect on functional and anatomical outcomes in cCSCR patients. The evidence presented is relevant to current management of the condition, but further studies on larger samples and longer treatment periods (> 3 months) are needed to obtain a better estimate of the effect of interventions.

## Supplementary Information

**Additional file 1**: Data Extraction

**Additional file 2:** Prisma 2020 Checklist

## Data Availability

Additional files.

## Abbreviations

CSCR: Central serous chorioretinopathy
PED: Pigment epithelial detachment
cCSCR: Chronic Central serous chorioretinopathy
SRF: Subretinal fluid
RPE: Retinal pigment epithelium
PDT: Photodynamic therapy
MRAs: Mineralocorticoid receptor antagonists
BCVA: Best-corrected visual acuity
RCT: Randomized clinical trials
ETDRS: Early Treatment Diabetic Retinopathy Study
CST: Central subfield thickness
SFH: Subretinal fluid height
CCT: Central choroidal thickness
OCT: Optical coherence tomography
MD: Mean difference
95% CI: 95% confidence intervals
SMD: Standardized mean difference
MCID: Minimal clinically important difference
