# Supplementary material for: Mineralocorticoid receptor antagonists for chronic central serous chorioretinopathy: systematic review and meta-analyses
Source: Int J Retina Vitreous. 2022 Jun 7;8:34. doi: 10.1186/s40942-022-00385-1 (PMC9172176; doi:10.1186/s40942-022-00385-1)
Supplement: Supplementary file 1 — Additional file 1: Data Extraction [file 40942_2022_385_MOESM1_ESM.pdf]

Study	Study design	Study grouping	Recruitment
Bousquet 2015	ECR	crossover	January 2012 to October 2012
Pichi 2016	ECR	crossover	May 2014 to November 2014
Schwartz 2017	ECR	parallel	August 2014 to May 2015
Rahimy 2018	ECR	parallel	December 2013 to December 2014
Lotery 2020	ECR	parallel	January 2017 to February 2018

Setting	Uni or multicentric
Ophthalmology Department (outpatient)	Uni
Multimedica SpA—San Giuseppe Hospital	Uni
Retina clinic at the Tel Aviv Medical Center	Uni
Retina Service of Wills Eye Hospital and the off	Uni
UK National Health Service secondary care hos	Multi (22)

Length of follow-up
1 month
1 month
6 months (treatment for 3 months)
9 weeks
12 months
